# Comparison of next-generation sequencing with traditional methods for pathogen detection in cases of lower respiratory tract infection at a community hospital in Eastern China

**DOI:** 10.1097/MD.0000000000032423

**Published:** 2022-12-23

**Authors:** Yi Yang, Xingxing Zhu, Yahong Sun, Kun Qian, Zhihao Liu

**Affiliations:** a Department of Respiratory Medicine, Haining People’s Hospital, Haining, China; b Haining People’s Hospital, Haining, China.

**Keywords:** lower respiratory tract infection, next-generation sequencing, pathogens

## Abstract

Lower respiratory tract infection (LRTI) is still a threat to human health. Metagenomics next-generation sequencing (NGS) provides an efficient and unbiased way to identify LRTI pathogens, and has been shown to have several advantages over traditional methods. However, its application is currently limited in low-resource settings. Our aim was to collect and analyze data on LRTI cases at a county-level community hospital in Eastern China over one year, in order to compare the efficiency of NGS and traditional methods including culture, nucleic acid amplification and antibody techniques. We performed NGS of bronchoalveolar lavage fluid (BALF) for pathogen identification in 71 patients with LRTI. We compared the detection rates, identified pathogens, and turnaround time of NGS with traditional methods. Pathogens were detected using traditional methods in 19 cases, and the results were compared with those obtained with the NGS technique in 60 cases. The pathogen detection rate of NGS (84.5%) was much higher than that of the traditional methods (26.8%). Moreover, with the traditional methods considered the gold standard, the consistency rate between NGS and traditional methods was 68.4%. For the 19 cases in which the traditional method was used, the main pathogens included invasive *Aspergillus* (5 cases), *Pseudomonas aeruginosa* (3 cases), *Candida albicans* (3 cases), and *Staphylococcus aureus* (2 cases). Among the 60 cases detected by NGS, the main pathogens included *Mycobacterium* (12 cases), *Streptococcus pneumoniae* (5 cases), *Klebsiella pneumoniae* (3 cases), *P. aeruginosa* (3 cases), *Haemophilus influenzae* (3 cases), and *S. aureus* (3 cases), *Aspergillus* (9 cases), *Pneumocystis jiroveci* (5 cases), *C. albicans* (3 cases), Human Papilloma Virus (9 cases), Epstein-Barr virus (8 cases), and parvovirus (6 cases). In addition, 2 cases of chlamydia and 1 case of mycoplasma infection were detected by NGS. The time taken to perform the NGS tests was significantly shorter than that taken with the traditional method. NGS analysis of bronchoalveolar lavage fluid, in combination with traditional pathogen detection methods, can improve the efficiency of pathogen detection. More attention should be paid to the regional epidemic characteristics of infectious pathogens in LRTI.

## 1. Introduction

Lower respiratory tract infection (LRTI) has one of the highest mortality rates worldwide, and it remains a threat to human health and is a heavy burden to the economy.^[1,2]^ With the aging of the Chinese population, the incidence of LRTI has been increasing, and previous data show that LRTI patients above 70 years old have a high mortality rate (about 3.72 per 100,000).^[3]^ If the patient’s condition cannot be controlled in an effective and timely manner, LRTI can progress to severe pneumonia, even septic shock and multiple organ dysfunction syndrome in some cases.^[4]^ Patients might die in some severe cases.

Traditionally, clinicians administer empirical antiinfection therapy,^[5]^ and simultaneously obtain sputum samples for smear and culture, based on the results of which physicians then administer targeted therapy. However, the pathogen identification rate by traditional methods, including culture, is low, and the turnaround time is considerable. In addition, viral pathogens cannot be detected by the traditional culture method, and the detection rate of viruses by nucleic acid amplification or antibody techniques is limited.^[6]^ As a result, the responsible pathogen cannot be detected in time, and patients develop resistance to antimicrobial drugs used in the empirical antiinfection treatment.^[7]^ Therefore, timely and effective pathogen detection methods are critical for improving the diagnosis rate and the effectiveness of targeted anti-infective therapy.^[8]^ The high-throughput sequencing technology metagenomics next-generation sequencing (NGS or mNGS) provides an efficient and unbiased way to identify pathogens in host-associated and environmental samples.^[9]^ The findings of proof-of-concept studies on NGS techniques have recently led to their application as a routine tool in the laboratory.^[9]^ However, despite growing evidence for the efficiency of NGS in the diagnosis of infectious diseases, community-level hospitals in China lack the resources required to implement NGS methods and still rely on traditional methods, such as culture and acid amplification techniques, as the first choice for the diagnosis of infectious pathogens.^[10,11]^

In the present study, we collected data on LRTI cases managed by our department in a county-level community hospital in Eastern China over one year, in order to summarize and analyze our experience with pathogen diagnosis, and to compare the traditional method and NGS with regard to their pathogen detection rates.

## 2. Methods

### 2.1. Patients

The study included patients with LRTI who were treated at the Department of Respiratory Medicine, Haining People’s Hospital, Zhejiang Province, Eastern China, between April 1, 2021, and March 31, 2022. The inclusion criteria were diagnosis of LRTI based on clinical manifestations and pulmonary imaging with high-resolution chest tomography (HRCT) at the outpatient department and failure to respond to initial empiric therapy. Based on clinical evidence, such as the discovery of new lesions by HRCT or changes in the patient’s general condition, bronchoalveolar lavage was carried out by bronchoscopy and bronchoalveolar lavage fluid (BALF) was collected for analysis. The following data were documented: name, gender, age, symptoms, co-existing diseases, and outcomes. Informed consent was obtained from the patients or their family members for the use of their information and specimens, and the study was approved by the hospital’s medical ethics committee.

### 2.2. Methods

Peripheral blood samples and BALF samples of the patients were collected. The following tests were performed on peripheral blood samples: routine whole blood analysis; respiratory virus screening; and analysis of high-sensitivity C-reactive protein, procalcitonin (PCT), and other indicators. Pathogen detection of the BALF samples was performed using traditional measurements and NGS. Once a BALF sample is collected, it sent for testing within 2 hours or stored at −20°C until transport. The turnaround time is defined as the duration between the time at which the samples are sent and the time at which the results are obtained.

### 2.3. NGS analysis

After bronchoalveolar lavage with fiberoptic bronchoscopy, sterile tubes were used to obtain BALF: 5 mL of the sample was collected in a 40-mL sterile tube and immediately stored at 4°C for transport. NGS detection was performed by Huada Gene Co. Ltd (Nanjing, Jiangsu Province, China). using the Q-mNGS quantitative method for metagenomic detection. The steps are as follows: BALF samples are added to the NGS master automated workstation for automated nucleic acid extraction, nucleic acid fragmentation, end blunting, end adenylation (addition of a single base A at the 3′ end), sequencing adapter ligation, and purification. The purified sequences formed the sequencing library, which was quantified with a real-time PCR instrument. Shotgun sequencing of the library was performed using the Illumina Nextseq high-throughput sequencing platform. Each library is expected to generate 20 million single-ended 75-bp sequences. Bioinformatics analysis was performed on the library sequence data. The human genome sequence data in it (GRCh38.p13) were filtered out, and the remaining sequence data were aligned with microbial reference databases (NCBI GenBank and in-house curated microbial genome data) to determine microbial species and relative abundance.

### 2.4. Statistical analysis

SPSS24.0 (IBM SPSS Statistics, Version 24.0. Armonk, NY) and Prism9 statistical software were used to analyze the data. Measurement data were expressed as mean ± standard deviation (x ± s) and analyzed with the *t* test, while count data were expressed as rate (%) and analyzed using the χ^2^ test. *P* values < .05 were considered to indicate statistically significant differences.

## 3. Results

### 3.1. General characteristics of the LRTI patients

A total of 71 patients were included in this study, including 46 male patients (64.8%) and 25 female patients (35.2%), with an age range of 18 to 92 years and average age of 60.7 years. At the time of consultation, it was confirmed that all the patients had abnormal lung imaging findings on chest HRCT. Among them, 50 (70.4%) patients presented with cough and sputum, 45 (63.4%) presented with fever (highest body temperature, 40.5°C), 13 (18.3%) presented with chest tightness and shortness of breath, 3 (4.2%) patients presented with hemoptysis, and the remaining 6 patients presented with other symptoms (e.g., chest pain). The general characteristics of the patients are presented in Table 1.

**Table 1:** General characteristics of patient population.

	Number (%)
Gender	Total 71 (100)
Male	46 (64.8)
Female	25 (35.2)
Age	years old
Range	18–92
Average	60.7
Clinical manifestation	
Abnormal radiography	71 (100)
Cough and expectoration	50 (70.4)
Fever	45 (63.4)
Tightness shortness of breath	13 (18.3)
Hemoptysis	3 (4.2)
Others (chest pain, etc.)	6 (8.5)

### 3.2. Pathogen detection

Pathogens were detected by traditional methods in 19 cases. These 19 cases included 1 case with positive acid-fast staining of the sputum smear that was confirmed as a *Mycobacterium tuberculosis* infection. No positive results were obtained with traditional methods in the remaining 52 cases, and the positive detection rate with traditional methods was 26.8% (19/71). Pathogens were detected in a total of 60 cases by the NGS method, and the remaining 11 cases tested negative. The positive detection rate with the NGS method was 84.5% (60/71). If the traditional method is assumed to be the gold standard, in 13 cases of the 19 cases with positive results, the same pathogen was detected by NGS. Of the remaining 6 cases, in 2 cases, the pathogens were not detected by NGS, and in the rest 4 cases, the detected pathogen was different from that determined with the NGS method. Thus, the NGS method and the traditional method exhibited 68.4% (13/19) consistency.

### 3.3. Distribution of detected pathogens

Among the 19 positive cases detected by the traditional method, the main pathogens included invasive *Aspergillus* (n = 5), *Pseudomonas aeruginosa* (n = 3), *Candida albicans* (n = 3, including 1 case caused by *C. albicans* and *C. glabrata* simultaneously), and *Staphylococcus aureus* (n = 2). No viral pathogens were detected. Except for the case with simultaneous *C. albicans* and *C. glabrata* infection, only one pathogen was detected in the remaining 18 cases.

**Figure 1.**
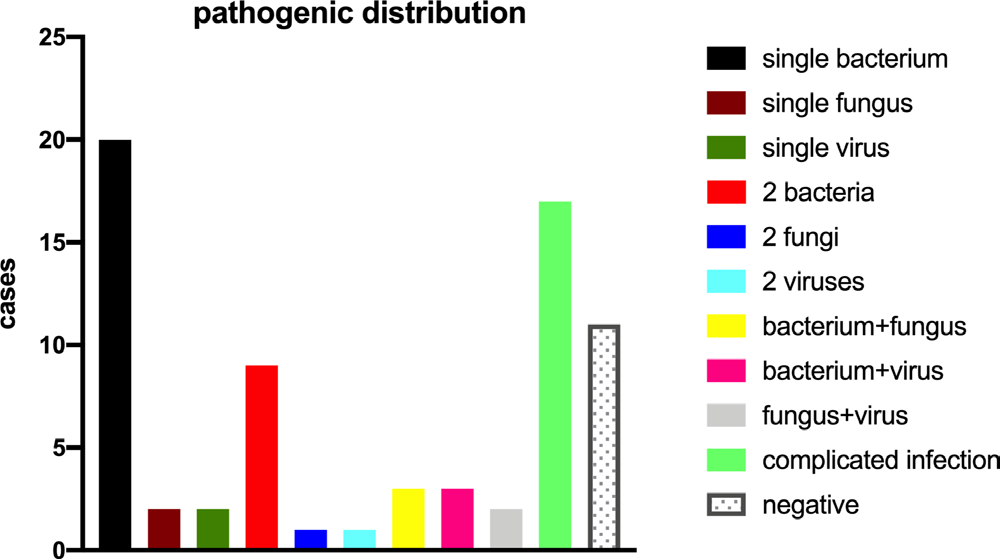
Distribution pattern of pathogens identified by the NGS technique. The graph depicts the type and number of pathogens detected. NGS = next-generation sequencing.

Among the 60 positive cases detected by the NGS method, a single bacterium was detected in 20 cases; a single fungus, in 2 cases; a single virus, in 2 cases; two different bacteria, in 9 cases; one bacterium and one fungus, in 3 cases; one bacterium and one virus, in 3 cases; two different fungi, in 1 case; one fungus and one virus, in 2 cases; two different viruses, in 1 case; mixed infection with 3 or more pathogens, in 17 cases (Fig. 1). Among the detected bacterial pathogens, *Mycobacterium* was detected in 12 cases; *Streptococcus pneumoniae*, in 5 cases; and *Klebsiella pneumoniae*, *P. aeruginosa*, *Haemophilus influenzae*, and *S. aureus*, in 3 cases. Among the fungal pathogens, the three most commonly detected ones were *Aspergillus* (n = 9), *Pneumocystis jiroveci* (n = 5), and *C. albicans* (n = 3). Among the viral pathogens, the three most commonly detected ones were Human Papilloma Virus (HPV, n = 9), Epstein–Barr virus (n = 8), and parvovirus (n = 6). The detected *Mycobacterium* species were further analyzed and identified as *M. tuberculosis* (n = 8), *M. avium* Chester (n = 2), *M. kansasii* (n = 1), and *M. abscessus* (n = 1). In addition, 2 cases of chlamydia infection and 1 case of mycoplasma infection were detected with NGS.

### 3.4. Turnaround time

**Figure 2.**
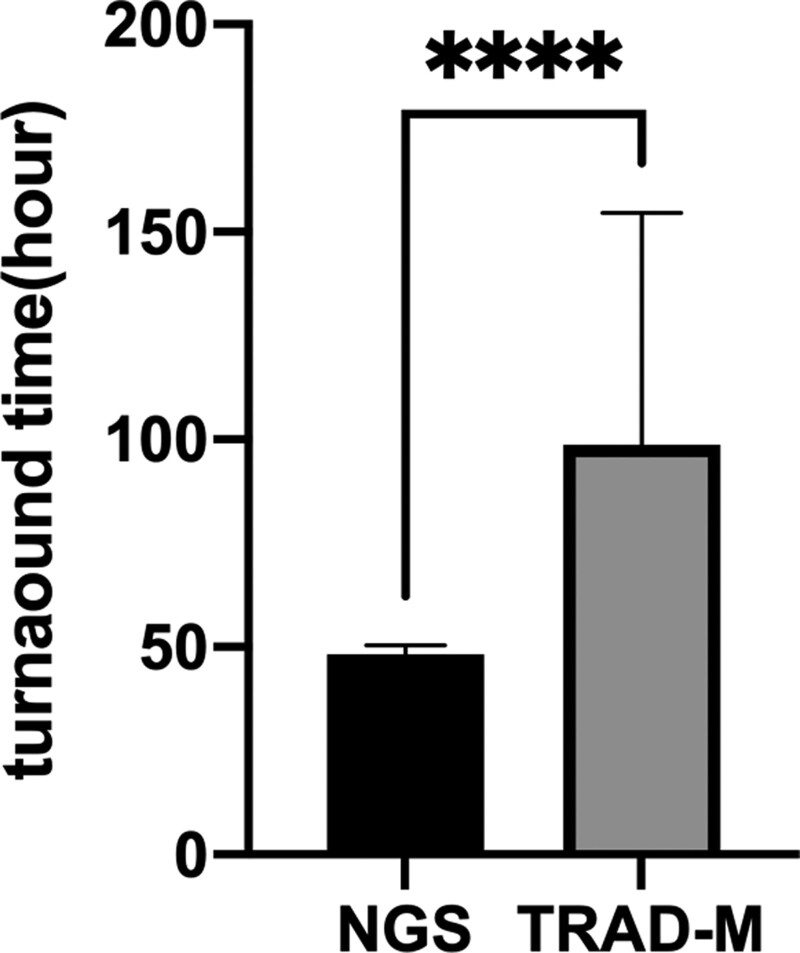
Comparison of the turnaround time of NGS and the traditional methods. As shown in the graph, the traditional methods had a significantly higher turnaround time than NGS. ****P < .0001. NGS = next-generation sequencing.

The longest turnaround time with the traditional method was about 193 hours, and the average turnaround time was about 98.8 hours. This means that the average turnaround time with the traditional method was about 4 days. The average turnaround time with the NGS method was about 48 hours or 2 days. Thus, there was a significant difference in the turnaround time of the two methods, with the NGS group having a significantly shorter turnaround time (Fig. 2).

### 3.5. Other clinical indicators

The peripheral blood WBC was in the range of 0.8 to 22.8 × 10^9^/L (average, 8.57 × 10^9^/L), and the neutrophil percentage was in the range of 2 % to 95.2% (average, 73.8%). The high-sensitivity C-reactive protein values were 0.2 to 233.7 mg/L (average, 76.8 mg/L). The cutoff value of PCT was defined as >0.5 ng/L. In 12 cases, the PCT values were more than 0.5 ng/L, and fungal infection was detected in these 12 cases by both methods. Of the 12 cases, 10 were positive and 2 were negative according to the NGS method, while 5 were positive and 7 were negative according to the traditional method. There was a significant difference in the positive detection rates of the two groups (Person χ^2^ test, *P* = .035).

### 3.6. Patient outcome

After the patients were diagnosed based on the NGS results, they were managed with anti-infection strategies specific to the detected pathogens. Most of the patients recovered, but 8 patients were transferred to the ICU for further treatment. Among the 8 patients, 5 were transferred back to the general ward, but 3 patients died. The treatment success rate was 95.8% (68/71).

## 4. Discussion

The results of the present study imply that NGS is an irreplaceable method for the diagnosis of infectious disease. The pathogen identification rate of the NGS method was 84.5%, which was significantly higher than the identification rate of 26.8% with the traditional method. Even when the traditional method was considered as the golden standard, the consistency between the NGS and the traditional method reached 68.4%, under circumstances where all the viral pathogens (which were identified with NGS but not with the traditional methods) were omitted. Further, in agreement with our findings, it has been demonstrated that NGS is not only far superior to traditional methods in terms of turnaround time, but also has unparalleled advantages for the diagnosis of multiple pathogens or complicated infections.^[12]^ Even though the adoption rate of NGS methods in low in regions of the world that are resource poor, the continuous advancements and marked drop in the cost of NGS diagnostic techniques^[13]^ are expected to lead to an increase in the use of NGS combined with traditional methods for pathogen detection in complicated infectious diseases.

PCT, a member of the calcitonin super family, is found at very low concentrations in plasma under normal physiological conditions. The rapid elevation in the concentration of PCT and other newly emerging biomarkers during an infection and its correlation with disease severity make them ideal biomarkers for bacterial infection.^[14,15]^ However, it is not clear whether PCT can be used to differentiate between fungal and bacterial infections.^[16]^ In invasive fungal infections, the serum level of PCT increases only slightly or does not increase obviously.^[17]^ However, in the present study, fungal infections were confirmed by both NGS and traditional methods in cases in which PCT was higher than the cutoff value for infection detection. Thus, our findings might imply that PCT could be used as an effective marker in invasive fungal infections. In addition, our results indicated that the NGS method was more efficient in detecting pathogens in cases with PCT greater than 0.5 ng/L. Thus, in cases in which the PCT level is higher than 0.5 ng/L and the pathogen cannot be clearly identified, the NGS method should be considered to assist in diagnosis.

In this study, LRTI cases that were treated at a county-level community hospital in Eastern China over a whole year were analyzed. Therefore, the findings may, to some extent, reflect the epidemic characteristics of LRTI at the regional community level. In a sense, the data may also reflect any significant changes in the causative pathogens of LRTI that occurred during the COVID-19 pandemic. From our findings, it could be inferred that the composition of LRTI pathogens is still dominated by bacteria or bacteria-based infections, and *S. pneumoniae* is still a common causative pathogen of community-acquired LRTI; this is consistent with the LRTI characteristics before the COVID-19 pandemic.^[18,19]^ However, it should be noted that the incidence of tuberculosis caused by *M. tuberculosis* has been gradually rising again. Among all the bacterial pathogens detected in this study, *Mycobacterium* had the highest detection rate and was identified in 12 cases, of which 8 were caused by *M. tuberculosis*. This is surprising, because national-level data indicate that the incidence of tuberculosis in China has been decreasing dramatically as a result of the continuous development of China’s economy, improvement in people’s living conditions and hygiene habits, and the promotion of anti-tuberculosis treatments.^[20–22]^ This implies that attention should be paid to the regional epidemic characteristics of infectious pathogens when physicians manage LRTI. However, this study has some defects, limited by the sample size and local patients’ resource, the presented result could reflect only a little scope of the eastern China LRTI pathogen distribution pattern, further data is impending needed. Moreover, among the detected fungal pathogens, *Aspergillus* and *P. jiroveci* were the two most commonly detected ones, as they were detected in 14 patients. Six of these fourteen patients had received long-term chemotherapy for malignant tumors, and one patient had been on long-term treatment with immunosuppressive drugs and glucocorticosteroids for systemic lupus erythematosus. Thus, in patients with malignant tumors or other immunodeficiency diseases who are receiving immunosuppressive treatment, it is necessary to consider infections caused by invasive fungal pathogens when considering the responsible pathogens.

## 5. Conclusion

The present findings imply that LRTI is still a common lethal disease in China, and the incidence and responsible pathogens may differ at the community level. Our findings also indicate that NGS analysis of bronchoalveolar lavage fluid, in combination with traditional methods, can significantly shorten the turnaround time for pathogen detection, improve the efficiency of pathogen detection, and improve the prognosis and survival of patients. Further, more attention should be paid to the regional epidemic characteristics of infectious pathogens when physicians manage LRTI cases.

## Author contributions

ZL conceived the idea. YY, XZ, YS, KQ collected the data. All authors analyzed and interpreted the data. ZL supervised the whole experiments. YY drafted the manuscript. All authors made critical revisions and approved the final version of the manuscript.

**Conceptualization:** Zhihao Liu.

**Data curation:** Yi Yang, Yahong Sun, Kun Qian.

**Formal analysis:** Xingxing Zhu.

**Funding acquisition:** Xingxing Zhu.

**Supervision:** Zhihao Liu.

**Writing – original draft:** Zhihao Liu.

**Writing – review & editing:** Zhihao Liu.

## References

[1] Liu W Wang W Liu J. Trend of mortality and years of life lost due to chronic obstructive pulmonary disease in China and its provinces, 2005-2020. Int J Chron Obstruct Pulmon Dis. 2021;16:2973–81. 34744434 10.2147/COPD.S330792 PMC8565891

[2] Mahashur A. Management of lower respiratory tract infection in outpatient settings: focus on clarithromycin. Lung India. 2018;35:143–9. 29487250 10.4103/lungindia.lungindia_262_17 PMC5846264

[3] Liu Q Jing W Liu M. Health disparity and mortality trends of infectious diseases in BRICS from 1990 to 2019. J Glob Health. 2022;12:04028. 35356649 10.7189/jogh.12.04028 PMC8943566

[4] Ljungström LR Jacobsson G Andersson R. Respiratory viral infections are underdiagnosed in patients with suspected sepsis. Eur J Clin Microbiol Infect Dis. 2017;36:1767–76. 28516200 10.1007/s10096-017-2990-z PMC5602075

[5] Blasi F Concia E Del Prato B On behalf of the *MASTER working group. The most appropriate therapeutic strategy for acute lower respiratory tract infections: a Delphi-based approach. J Chemother. 2017;29:274–86. 28298164 10.1080/1120009X.2017.1291467

[6] Li N Ma X Zhou J. Clinical application of metagenomic next-generation sequencing technology in the diagnosis and treatment of pulmonary infection pathogens: a prospective single-center study of 138 patients. J Clin Lab Anal. 2022:e24498. 35622934 10.1002/jcla.24498 PMC9279992

[7] Liu Y Zhang Y Zhao W. Pharmacotherapy of lower respiratory tract infections in elderly-focused on antibiotics. Front Pharmacol. 2019;10:1237. 31736751 10.3389/fphar.2019.01237 PMC6836807

[8] Liu J Qi J Chen W. Multi-branch fusion auxiliary learning for the detection of pneumonia from chest X-ray images. Comput Biol Med. 2022;147:105732. 35779478 10.1016/j.compbiomed.2022.105732 PMC9212341

[9] Song S Ma L Xu X. Rapid screening and identification of viral pathogens in metagenomic data. BMC Med Genomics. 2021;14(Suppl 6):289. 34903237 10.1186/s12920-021-01138-z PMC8668262

[10] Zhang X Li Y Yin J. Application of next-generation sequencing in infections after allogeneic haematopoietic stem cell transplantation: a retrospective study. Front Cell Infect Microbiol. 2022;12:888398. 35774403 10.3389/fcimb.2022.888398 PMC9239075

[11] Tsang HF Yu ACS Jin N. The clinical application of metagenomic next-generation sequencing for detecting pathogens in bronchoalveolar lavage fluid: case reports and literature review. Expert Rev Mol Diagn. 2022;22:575–82. 35473493 10.1080/14737159.2022.2071607

[12] Indelli PF Ghirardelli S Violante B. Next generation sequencing for pathogen detection in periprosthetic joint infections. EFORT Open Rev. 2021;6:236–44. 34040801 10.1302/2058-5241.6.200099 PMC8142595

[13] Ramachandran PS Wilson MR. Metagenomics for neurological infections - expanding our imagination. Nat Rev Neurol. 2020;16:547–56. 32661342 10.1038/s41582-020-0374-y PMC7356134

[14] Vijayan AL Vanimaya Ravindran S. Procalcitonin: a promising diagnostic marker for sepsis and antibiotic therapy. J Intensive Care. 2017;5:51. 28794881 10.1186/s40560-017-0246-8 PMC5543591

[15] Azzini AM Dorizzi RM Sette P. A 2020 review on the role of procalcitonin in different clinical settings: an update conducted with the tools of the evidence based laboratory medicine. Ann Transl Med. 2020;8:610. 32566636 10.21037/atm-20-1855 PMC7290560

[16] Cortegiani A Misseri G Ippolito M. Procalcitonin levels in candidemia versus bacteremia: a systematic review. Crit Care. 2019;23:190. 31138262 10.1186/s13054-019-2481-y PMC6537202

[17] Stoma I Karpov I Uss A. Low levels of procalcitonin or presepsin combined with significantly elevated c-reactive protein may suggest an invasive fungal infection in hematological patients with febrile neutropenia. Hemasphere. 2019;3:e170. 31723809 10.1097/HS9.0000000000000170 PMC6745942

[18] Zhu YG Tang XD Lu YT. Contemporary situation of community-acquired pneumonia in China: a systematic review. J Transl Int Med. 2018;6:26–31. 29607301 10.2478/jtim-2018-0006 PMC5874484

[19] Qin T Zhou H Ren H. Incidence, etiology, and environmental risk factors of community-acquired pneumonia requiring hospitalization in China: a 3-year, prospective, age-stratified, multicenter case-control study. Open Forum Infect Dis. 2021;8:ofab499. 35548172 10.1093/ofid/ofab499 PMC8522381

[20] Ding C Wang S Shangguan Y. Epidemic trends of tuberculosis in China from 1990 to 2017: evidence from the global burden of disease study. Infect Drug Resist. 2020;13:1663–72. 32606817 10.2147/IDR.S249698 PMC7293403

[21] Long Q Guo L Jiang W. Ending tuberculosis in China: health system challenges. Lancet Public Health. 2021;6:e948–53. 34838198 10.1016/S2468-2667(21)00203-6

[22] Yu S Ma J Jia Z. Estimating the incidence of tuberculosis - Shanghai, China, 2025-2050. China CDC Wkly. 2020;2:995–8. 34594823 10.46234/ccdcw2020.266 PMC8422224

